# Supplementary material for: Enhancing secondary school students’ science process skills through guided inquiry-based laboratory activities in biology
Source: PLoS One. 2025 Apr 10;20(4):e0320692. doi: 10.1371/journal.pone.0320692 (PMC11984731; doi:10.1371/journal.pone.0320692)
Supplement: SI File — This appendix includes the SPS essay test provided to students, detailing sample experimental topics, instructions, and essay questions designed to assess students’ science process skills in biology. SI Appendix-B). SPS Essay Test RUBRIC. This rubric outlines the scoring criteria for evaluating students’ responses to the SPS essay test, including indicators for formulating hypotheses, designing experiments, interpreting data, and drawing conclusions. (DOCX) [file pone.0320692.s001.docx]

**Appendix A: Science Process Skills (SPS) essay test**

Dear students

I am conducting a PhD dissertation research. This test is intended to find your science process skills. The information obtained from this test will serve as an input in enhancing students’ biology learning. The worksheet test consists of 6 sample experimental topics and 8 essay questions. Thus, you are asked to design and conduct one of the experimental topics you will select in individually. Moreover, you are also requested to give your genuine response individually to the essay questions on the space provided. The responses to the questions will be kept confidential.

Thank you for your cooperation!

Section A: Background Information

1. Full Name____________________________________________________
2. Role No___________
3. Gender: Put a tick mark

Male Female

1. Grade &Section ____________
2. Name of School __________________________________________________

**Section B: Sample experimental topics and students’ worksheet questions**

Instruction: Select a project/experiment topic and design an experiment that you can complete with materials/ equipment and reagents found in your laboratory/environment. Then complete the following worksheet (or answer the questions on a separate sheet of paper) outlining your experiment and discussing your results individually.

**Sample project/experimental topics**

- Testing a leaf for starch
- Showing that oxygen is produced during photosynthesis
- Showing that chlorophyll is needed for photosynthesis
- Looking at the stomata in a leaf
- Investigating factors which affect transpiration using potometer.
- Investigation of the role of the shoot tip in the growth of a plant

1. **Question/Purpose**_________________________________________________________________________________________.
2. **Hypothesis**_________________________________________________________________________________________.
3. **Independent Variable**​ (What I am changing on purpose?)
   __________________________________________________________________________.
4. **Dependent Variable**​ (What am I measuring that responds to the independent variable?)

**__________________________________________________________________________.**

1. **Materials**​/**equipment & Reagents** (List everything you need)

**__________________________________________________________________________________.**

1. **Procedure**​ (Write step by step, in order, what you will do for this experiment – repeatable by someone else)
   ________________________________________________________________________________________.
2. **Interpreting the data** (List all information collected during your experiment. Organize, interpret and present the data clearly. (Tables and graphs can be used.)

**______________________________________________________.**

1. **Conclusion**​(What happened? Did your results support your conclusion? What was surprising? What did you learn?)
   ____________________________________________________________________.

**Appendix B: SPS essay test RUBRIC**

Full Name____________________________Grade& Section__________Rol. No._______

School________________

| No | SPS | SPS indicators | Score |
| --- | --- | --- | --- |
| 1 | Formulating hypothesis | 1. Formulating rational/relevant hypothesis | Score 4 if 4 indicators are met  Score 3 if 3 indicators are met  Score 2 if 2 indicators are met  Score 1 if 1 indicators are met  Score 0 if 0 indicators are met |
|  |  | 1. Hypotheses that use concepts, theories and laws |  |
|  |  | 1. Hypotheses are in accordance with the purpose of the experiment |  |
|  |  | 1. Using correct and logical language |  |
| 2 | Designing an experiment | 1. The student is able to find suitable materials, equipment and reagents with the experiments | Score 4 if 4 indicators are met  Score 3 if 3 indicators are met  Score 2 if 2 indicators are met  Score 1 if 1 indicators are met  Score 0 if 0 indicators are met |
|  |  | 1. Independent and dependent variables are clearly identified. |  |
|  |  | 1. Able to make systematic and coherent experimental procedures |  |
|  |  | 1. The experiment tests what it should |  |
| 3 | Interpreting data | 1. Listing all information collected during the experiment | Score 4 if 4 indicators are met  Score 3 if 3 indicators are met  Score 2 if 2 indicators are met  Score 1 if 1 indicators are met  Score 0 if 0 indicators are met |
|  |  | 1. Combining all information from various theories with the experiment results |  |
|  |  | 1. Connecting between variables |  |
|  |  | 1. Organize, interpret and present the data clearly (e.g. Tables and graphs are used.) |  |
| 4 | Drawing conclusion | 1. Related to the experimental hypotheses and question | Score 4 if 4 indicators are met  Score 3 if 3 indicators are met  Score 2 if 2 indicators are met  Score 1 if 1 indicators are met  Score 0 if 0 indicators are met |
|  |  | 1. Linking tendency between variables |  |
|  |  | 1. Conclusions are in accordance with the experimental results |  |
|  |  | 1. Accurate and supported by the data analysis |  |
