# Supplementary material for: Enhancing secondary school students’ science process skills through guided inquiry-based laboratory activities in biology
Source: PLoS One. 2025 Apr 10;20(4):e0320692. doi: 10.1371/journal.pone.0320692 (PMC11984731; doi:10.1371/journal.pone.0320692)
Supplement: S2 File — (DOCX) [file pone.0320692.s002.docx]

| SPS Research Data for Manuscript number: PONE-D-24-48585R1. The dataset can be accessed using the DOI: 10.1371/journal.pone.0320692 | | | | | | | | | | | | | |
| --- | --- | --- | --- | --- | --- | --- | --- | --- | --- | --- | --- | --- | --- |
| Ser. No | Respondents | Gender | Groups | Form Hypothesis pretest | Design Experiment pretest | interpret Data pretest | Drawing Conclusion pretest | Form Hypothesis post-test | Design Experiment posttest | Interpret Data post-test | Drawing Conclusion post-test | SPS pre-test total (out of 16) | SPS post- test total (out of 16) |
| 1 | FSS4 | Female | EG | 1 | 1 | 1 | 0 | 0 | 0 | 0 | 0 | 3 | 0 |
| 2 | FSS6 | Female | EG | 2 | 0 | 0 | 0 | 2 | 3 | 4 | 3 | 2 | 12 |
| 3 | FSS7 | Female | EG | 0 | 0 | 0 | 0 | 0 | 0 | 0 | 0 | 0 | 0 |
| 4 | FSS8 | Female | EG | 2 | 1 | 0 | 0 | 1 | 2 | 1 | 3 | 3 | 7 |
| 5 | FSS11 | Female | EG | 0 | 0 | 0 | 0 | 0 | 1 | 0 | 0 | 0 | 1 |
| 6 | FSS12 | Female | EG | 0 | 0 | 0 | 0 | 0 | 0 | 0 | 0 | 0 | 0 |
| 7 | FSS14 | Female | EG | 0 | 0 | 2 | 0 | 0 | 2 | 0 | 0 | 2 | 2 |
| 8 | FSS18 | Female | EG | 2 | 0 | 1 | 0 | 0 | 0 | 0 | 0 | 3 | 0 |
| 9 | FSS19 | Female | EG | 0 | 1 | 0 | 0 | 2 | 3 | 3 | 2 | 1 | 10 |
| 10 | FSS 20 | Female | EG | 0 | 0 | 0 | 0 | 2 | 1 | 1 | 0 | 0 | 4 |
| 11 | FSS 21 | Female | EG | 1 | 0 | 2 | 0 | 2 | 0 | 0 | 0 | 3 | 2 |
| 12 | FSS 22 | Female | EG | 0 | 1 | 1 | 0 | 2 | 1 | 0 | 0 | 2 | 3 |
| 13 | FSS 24 | Female | EG | 1 | 1 | 2 | 0 | 4 | 3 | 4 | 2 | 4 | 13 |
| 14 | FSS25 | Female | EG | 2 | 0 | 0 | 0 | 2 | 1 | 0 | 0 | 2 | 3 |
| 15 | FSS28 | Female | EG | 3 | 1 | 1 | 0 | 4 | 3 | 2 | 3 | 5 | 12 |
| 16 | FSS29 | Female | EG | 2 | 0 | 2 | 0 | 2 | 0 | 0 | 1 | 4 | 3 |
| 17 | FSS30 | Female | EG | 2 | 1 | 1 | 0 | 2 | 3 | 3 | 3 | 4 | 11 |
| 18 | FSS32 | Female | EG | 2 | 0 | 1 | 0 | 0 | 1 | 0 | 2 | 3 | 3 |
| 19 | FSS33 | Female | EG | 1 | 0 | 0 | 0 | 1 | 2 | 3 | 4 | 0 | 10 |
| 20 | FSS34 | Female | EG | 1 | 0 | 0 | 0 | 0 | 0 | 0 | 0 | 1 | 0 |
| 21 | FSS42 | Female | EG | 3 | 2 | 2 | 0 | 1 | 1 | 1 | 0 | 7 | 3 |
| 22 | FSS43 | Female | EG | 2 | 2 | 2 | 0 | 2 | 1 | 0 | 0 | 6 | 3 |
| 23 | FSS44 | Female | EG | 2 | 1 | 0 | 0 | 0 | 0 | 0 | 0 | 3 | 0 |
| 24 | FSS46 | Female | EG | 2 | 2 | 0 | 0 | 4 | 3 | 0 | 1 | 4 | 8 |
| 25 | FSS1 | Male | EG | 0 | 1 | 0 | 0 | 0 | 0 | 0 | 0 | 1 | 0 |
| 26 | FSS2 | Male | EG | 3 | 2 | 1 | 0 | 3 | 2 | 2 | 0 | 6 | 7 |
| 27 | FSS3 | Male | EG | 1 | 1 | 0 | 0 | 1 | 1 | 0 | 0 | 2 | 2 |
| 28 | FSS5 | Male | EG | 1 | 0 | 0 | 0 | 2 | 0 | 0 | 0 | 1 | 2 |
| 29 | FSS9 | Male | EG | 1 | 1 | 1 | 0 | 2 | 0 | 0 | 0 | 3 | 2 |
| 30 | FSS10 | Male | EG | 1 | 2 | 1 | 1 | 3 | 2 | 3 | 4 | 5 | 12 |
| 31 | FSS13 | Male | EG | 3 | 1 | 2 | 0 | 4 | 3 | 2 | 3 | 6 | 12 |
| 32 | FSS15 | Male | EG | 2 | 1 | 0 | 0 | 4 | 3 | 2 | 3 | 3 | 12 |
| 33 | FSS16 | Male | EG | 2 | 1 | 0 | 0 | 4 | 4 | 0 | 2 | 3 | 10 |
| 34 | FSS17 | Male | EG | 1 | 0 | 1 | 0 | 4 | 3 | 3 | 3 | 2 | 13 |
| 35 | FSS 23 | Male | EG | 2 | 1 | 0 | 0 | 0 | 1 | 0 | 0 | 3 | 4 |
| 36 | FSS26 | Male | EG | 0 | 2 | 0 | 2 | 3 | 1 | 1 | 0 | 4 | 5 |
| 37 | FSS27 | Male | EG | 1 | 1 | 0 | 0 | 0 | 0 | 0 | 0 | 2 | 0 |
| 38 | FSS31 | Male | EG | 1 | 2 | 1 | 0 | 3 | 3 | 4 | 4 | 4 | 14 |
| 39 | FSS35 | Male | EG | 2 | 1 | 1 | 0 | 4 | 3 | 2 | 3 | 4 | 12 |
| 40 | FSS36 | Male | EG | 1 | 1 | 0 | 0 | 2 | 2 | 0 | 0 | 2 | 4 |
| 41 | FSS37 | Male | EG | 2 | 2 | 1 | 0 | 1 | 1 | 1 | 0 | 5 | 3 |
| 42 | FSS38 | Male | EG | 0 | 1 | 0 | 0 | 2 | 2 | 1 | 0 | 1 | 5 |
| 43 | FSS39 | Male | EG | 2 | 1 | 1 | 0 | 0 | 1 | 1 | 0 | 4 | 2 |
| 44 | FSS40 | Male | EG | 1 | 1 | 0 | 0 | 2 | 0 | 0 | 0 | 2 | 2 |
| 45 | FSS41 | Male | EG | 1 | 0 | 0 | 0 | 4 | 3 | 3 | 3 | 1 | 13 |
| 46 | FSS45 | Male | EG | 2 | 1 | 0 | 0 | 2 | 1 | 0 | 0 | 3 | 3 |
| 47 | AASS1 | Female | CG | 1 | 0 | 0 | 0 | 2 | 1 | 0 | 0 | 1 | 3 |
| 48 | AASS2 | Female | CG | 2 | 1 | 0 | 0 | 0 | 1 | 0 | 0 | 3 | 1 |
| 49 | AASS4 | Female | CG | 0 | 0 | 0 | 0 | 0 | 0 | 0 | 0 | 0 | 0 |
| 50 | AASS7 | Female | CG | 0 | 0 | 0 | 0 | 0 | 1 | 0 | 0 | 0 | 1 |
| 51 | AASS12 | Female | CG | 2 | 0 | 3 | 0 | 2 | 1 | 1 | 0 | 5 | 4 |
| 52 | AASS13 | Female | CG | 1 | 1 | 0 | 0 | 0 | 0 | 0 | 0 | 2 | 0 |
| 53 | AASS14 | Female | CG | 0 | 1 | 0 | 0 | 0 | 1 | 0 | 0 | 1 | 1 |
| 54 | AASS15 | Female | CG | 2 | 2 | 1 | 0 | 2 | 1 | 0 | 0 | 5 | 3 |
| 55 | AASS16 | Female | CG | 1 | 1 | 0 | 0 | 2 | 1 | 0 | 2 | 2 | 5 |
| 56 | AASS17 | Female | CG | 2 | 0 | 0 | 0 | 1 | 1 | 0 | 0 | 2 | 2 |
| 57 | AASS18 | Female | CG | 1 | 1 | 1 | 0 | 2 | 1 | 0 | 1 | 3 | 4 |
| 58 | AASS20 | Female | CG | 2 | 0 | 0 | 0 | 2 | 1 | 0 | 0 | 2 | 3 |
| 59 | AASS21 | Female | CG | 2 | 1 | 0 | 0 | 0 | 0 | 0 | 0 | 3 | 0 |
| 60 | AASS23 | Female | CG | 0 | 0 | 0 | 0 | 2 | 2 | 0 | 0 | 0 | 4 |
| 61 | AASS25 | Female | CG | 2 | 0 | 0 | 0 | 0 | 0 | 0 | 0 | 2 | 0 |
| 62 | AASS28 | Female | CG | 1 | 1 | 0 | 0 | 2 | 0 | 0 | 0 | 2 | 2 |
| 63 | AASS3 | Male | CG | 2 | 0 | 0 | 0 | 2 | 1 | 0 | 0 | 2 | 3 |
| 64 | AASS5 | Male | CG | 0 | 1 | 1 | 0 | 1 | 0 | 0 | 0 | 2 | 1 |
| 65 | AASS6 | Male | CG | 1 | 1 | 0 | 0 | 2 | 1 | 0 | 1 | 2 | 4 |
| 66 | AASS8 | Male | CG | 0 | 0 | 0 | 0 | 0 | 0 | 0 | 0 | 0 | 0 |
| 67 | AASS9 | Male | CG | 3 | 0 | 0 | 0 | 0 | 1 | 0 | 0 | 3 | 1 |
| 68 | AASS10 | Male | CG | 0 | 0 | 1 | 0 | 0 | 0 | 0 | 0 | 1 | 0 |
| 69 | AASS11 | Male | CG | 3 | 1 | 1 | 0 | 4 | 0 | 0 | 0 | 5 | 4 |
| 70 | AASS19 | Male | CG | 3 | 1 | 1 | 0 | 0 | 0 | 0 | 0 | 5 | 0 |
| 71 | AASS22 | Male | CG | 1 | 1 | 0 | 0 | 2 | 2 | 0 | 0 | 2 | 4 |
| 72 | AASS24 | Male | CG | 1 | 0 | 0 | 0 | 0 | 0 | 0 | 0 | 1 | 0 |
| 73 | AASS26 | Male | CG | 2 | 1 | 2 | 0 | 0 | 0 | 0 | 0 | 5 | 0 |
| 74 | AASS27 | Male | CG | 1 | 1 | 0 | 0 | 2 | 0 | 0 | 0 | 2 | 2 |
| 75 | AASS29 | Male | CG | 3 | 0 | 0 | 0 | 0 | 0 | 0 | 0 | 3 | 0 |
