# Supplementary material for: Enhancing secondary school students’ science process skills through guided inquiry-based laboratory activities in biology
Source: PLoS One. 2025 Apr 10;20(4):e0320692. doi: 10.1371/journal.pone.0320692 (PMC11984731; doi:10.1371/journal.pone.0320692)
Supplement: Attachment — Submitted filename: comments.docx [file pone.0320692.s003.docx]

**Enhancing Secondary School Students’ Science Process Skills through Guided Inquiry-based Laboratory activities in Biology**

**Comments**

1. “When laboratory experiments are used as an instructional strategy and students are actively engaged, most studies have found a significant improvement in students' achievement and SPS (9). Therefore, active learning techniques, such as inquiry-based learning, should replace traditional lecturing and cookbook-style laboratories”

If this has already been established, then what is the rational or basis for the current work?

2. Please provide justification for the selected biology topic
