# Supplementary material for: Enhancing secondary school students’ science process skills through guided inquiry-based laboratory activities in biology
Source: PLoS One. 2025 Apr 10;20(4):e0320692. doi: 10.1371/journal.pone.0320692 (PMC11984731; doi:10.1371/journal.pone.0320692)
Supplement: Attachment — Submitted filename: Response to Reviewers.docx [file pone.0320692.s005.docx]

Date: January 20, 2025

**Dear Mc Rollyn Daquiado Vallespin** **(**Academic Editor)

**PLOS ONE**

Subject: **Report on the incorporation of comments and suggestions**

Following our submission of the manuscript for publication we have received invaluable comments and suggestions that have improved the quality of the manuscript. We acknowledge the contributions of the editorial team and the reviewers for valuing our work and allowing us to improve the quality standard of the manuscript. We have incorporated all the comments/suggestions provided by the reviewers and the response on how we incorporated the comments is annexed below. Thank you again for your consideration!

With Best Regards

| **Content** | **Suggestions/comments** | **Response (ways how they are addressed)** |
| --- | --- | --- |
| **Abstract** | **Reviewer 1:**   i. The first sentence of the abstract presents the research objective, while the second and third sentences provide the research background. It would be more effective to present the background information first, followed by the objective.   **Reviewer 2:**   i. Refine the aim for greater clarity by explicitly linking it to the educational challenges mentioned (e.g., resource constraints, traditional methods). For example: "This study aims to evaluate the effectiveness of a Guided Inquiry-Based Laboratory Experiments Enriched Instructional (GIBLEI) approach in overcoming traditional teaching limitations and enhancing science process skills (SPS) among Ethiopian secondary school biology students." ii. Add "secondary school students" to the list of keywords to improve discoverability. | i. We completely accepted the comments, and we revised the abstract based on the given comments. We have highlighted the revised text in bright green color. i. We accepted the comment and tried to clarify the research aim by linking it to the educational challenges. Moreover, we highlighted the revision with dark yellow color. ii. We accepted the suggestion and the phrase "secondary school students" in to key words. The change was highlighted with green color. |
| **Introduction** | **Reviewer 1:**   i. The state-of-the-art section is weak due to the limited discussion of previous studies. As a result, the articulation of the research gap and the novelty is not strong enough.   **Reviewer 2:**   i. Clearly frame the problem statement and expand the significance of the study to connect the findings to broader implications for Ethiopian education and global education research. ii. Acknowledge the small sample size (N = 75, with only 29 in the control group) and discuss its implications for statistical power and generalizability.  - Provide a rationale for conducting the study, particularly given existing findings that inquiry-based learning improves SPS. Explain how the current work builds on or differs from past studies.   **Reviewer 3:**   i. Active learning techniques, such as inquiry-based learning, should replace traditional lecturing and cookbook-style laboratories. If this has already been established, then what is the rational or basis for the current work? | By accepting the suggestions:   i. We have tried to incorporate enough previous relevant studies in to the introduction part to enhance the articulation of the research gap and strengthen the novelty of the research. For instance, the introduction provides a solid overview of the literature related to Science Process Skills (SPS), inquiry-based learning (IBL), and the challenges faced in Ethiopian secondary schools. Moreover, the discussion expanded to clarify the specific research gap and the novelty of the current work. The purple color indicates some of the modification we have made. i. We have tried to clarify the problem statement and the significance of the study in the introduction section. The blue color indicates some of the modification we have made. ii. We accepted the comment and acknowledged in the small sample size in the limitation sections. The change was highlighted with light green color.  - We completely accepted the comment and included the rationale for conducting the study and how the current work builds on or differs from past studies. Some of the changes were highlighted with yellow color.  i. We accepted the comment and revised the content by completely removing the vague sentence. |
| **Methods** | **Reviewer 1:**   i. In the research design section, provide justification or evidence that both groups have similar characteristics and clarify what specific characteristics are deemed similar. ii. In the data analysis section, explain why the two different analyses (Welch's t-test and ANCOVA) to compare the performance of students in the two groups instead of opting for one consistent method. iii. Regarding gender: How is it positioned in the study? Why did you compare the performance of male and female students? If gender is treated as an independent variable, why did you not employ a two-way ANCOVA?   **Reviewer 2:**   i. Indicate whether the teachers' prior experience with inquiry-based methods was assessed, as this could influence the intervention's effectiveness. ii. Justify why eight weeks was chosen as the intervention duration and discuss whether this aligns with previous research or practical constraints. | By accepting the suggestions/Comments:   i. We included the features of the schools and the profiles of teachers in the sampling section and highlighted with yellow color (*These schools were chosen based on their similarity in facilities, including laboratory chemicals and equipment, library resources, ICT infrastructure, and the qualifications and experience of teachers*). ii. We accepted the comment and we gave reason for simultaneous use of Welch's t-test and ANCOVA in the data analysis section. We also highlighted the changes with turquoises color. iii. In this study, the role of gender was a secondary analysis to determine whether the intervention effects varied between male and female students, providing additional insights into the study’s findings. It is highlighted with pink color in the data analysis section. i. The teachers’ prior experience with inquiry-based methods was not formally assessed. However, the researchers addressed this potential variability through initial training and ongoing support throughout the study. The continuous guidance provided to both groups aimed to ensure that all teachers were adequately prepared to deliver the instructional approaches, thereby enhancing the consistency and reliability of the intervention outcomes. This was included in the treatment procedure section and highlighted with pink color. ii. We accepted the comment and we have included the justification in the treatment procedure section. The changes were highlighted with dark yellow color. |
| **Results** | **Reviewer 2:**   i. Provide clear and descriptive captions for all tables, ensuring each includes:  - A brief explanation of the table's content. - The statistical test performed (e.g., t-test, ANCOVA). - The significance level (e.g., p<0.05p < 0.05). | i. We accepted the comments and we provided clear and descriptive captions for all the tables according to the comments. |
| **Discussion** | **Reviewer 1:**   i. The discussion is too brief, comprising only two paragraphs. It is also inadequately supported by references, with only one scientific citation in the first paragraph. ii. The discussion primarily focuses on interpreting the analytical results and comparing findings with previous studies.     a. The manuscript lacks a deeper exploration of why the treatment yielded significant effects on students’ competencies.     b. A more thorough analysis and discussion of the mechanisms of the findings are necessary.     c. The implications of the findings are also important.   **Reviewer 2:**   i. Delve deeper into why GIBLEI was particularly effective, exploring the role of active learning or student engagement in fostering SPS. ii. Discuss how GIBLEI could be adapted for resource-limited settings iii. Provide practical recommendations for implementation. | i. We accepted the comments. Moreover, we revised and supported the discussion with more relevant references. ii. We completely accepted the comment. Thus;      a. The treatment yielded significant effects on students’ competencies due to the nature the GIBLEI. This was highlighted with bright green color in the discussion section. b. We also added the underlying mechanism behind the effectiveness of GIBLEI in discussion sections. It was also highlighted with blue color in the discussion sections. c. Moreover, the implications of the findings were also included in the conclusion and recommendation sections. The implications were highlighted with teal color. i. We completely accepted the comment and incorporated the reason why GIBLEI approach was effective in developing students SPS. This was highlighted with bright green color. ii. The mechanisms by which the GIBLEI could be adapted for resource-limited settings was discussed in the last paragraph of the discussion section and highlighted with pink color. iii. The practical recommendations for implementation of the GIBLEI was provided in the last paragraph of the conclusion and recommendation section and highlighted with yellow color. |
